# Genotype–phenotype correlation in patients with deletional and nondeletional mutations of Hb H disease in Southwest of Iran

**DOI:** 10.1038/s41598-022-08986-4

**Published:** 2022-03-22

**Authors:** Mohammad Hamid, Bijan keikhaei, Hamid Galehdari, Alihossein Saberi, Alireza Sedaghat, Gholamreza Shariati, Marziye Mohammadi-Anaei

**Affiliations:** 1 Department of Molecular Medicine, Biotechnology Research Center, Pasteur Institute of Iran, Tehran, Iran; 2 Research Center for Thalassemia and Hemoglobinopathy, Health Institute, Ahvaz Jundishapur University of Medical Sciences, Ahvaz, Iran; 3 Department of Genetics, Faculty of Sciences, Shahid Chamran University of Ahvaz, Ahvaz, Iran; 4 Department of Medical Genetic, Faculty of Medicine, Ahvaz Jundishapur University of Medical Sciences, Ahvaz, Iran; 5 Department of Endocrinology, Ahvaz Jundishapur University of Medical Sciences, Ahvaz, Iran; 6 Narges Medical Genetics and PND Laboratory, Ahvaz, Iran

**Keywords:** Genetics, Molecular medicine, Haematological diseases

## Abstract

We studied the alpha-globin gene genotypes, hematologic values, and transfusion-dependence of patients with Hb H disease. Molecular characterization of alpha-thalassemia was performed. We identified 120 patients with Hb H disease. Of these patients, 35 (29.16%) had deletional form of Hb H disease, and 85 (70.83%) had different form of non-deletional Hb H disease. The most frequently observed Hb H genotypes were --^Med^/–α^3.7^ in 33 patients (27.5%), α^CD19(-G)^ α^/αCD19(-G)^ α in 25 cases (20.83%), α^polyA2^α/α^polyA2^α in 15 (12.5%), and α^polyA1^α/α^polyA1^α in 13 (10.83%) respectively. The probability of receiving at least one transfusion blood in deletional form was observed in 3 of 35 (8.57%) patients which just seen in 3 of 33 (9%) patients with --^Med^/–α^3.7^ genotype. This form was also observed in 8 of 85 (9.4%) patients in non-deletional Hb H diseases which five of them had Med deletion in compound with alpha globin point mutations. Nondeletional Hb H disease was more severe than deletional Hb H disease requiring more blood transfusions. We can recommend that Med deletion in compound with alpha-globin point mutations, polyA1 and constant spring in homozygous form needs to be taken into consideration when offering counseling to high-risk couples.

## Introduction

Alpha thalassemia is one of the most common monogenic disorders in the Mediterranean region, Middle East and East and Southeast Asia, as well as in countries with migration from these regions^1,2^. The clinical manifestations of alpha globin abnormalities vary from the silent carrier state, in which only one α-globin gene is deleted, to fatal hydrops fetalis, in which all four α-globin genes are missing^3^. Hemoglobin H (Hb H) disease is caused by the loss of three α-globin genes, when there is only one functional alpha globin gene the patient produce a form of hemoglobin (Hb) composed of four β-chain (β4) called Hb H^4^. The clinical severity of patients with Hb H disease is variable, even in the presence of similar genotype, which is probably due to genetic and environmental modifiers^5^. More than 95% of α-thalassemia syndromes are caused by deletional abnormalities while the rest result from point mutations^6^.

Hb H patients also have been classified as mild, intermediate or severe phenotypes. Mild phenotype included the patients, who diagnosed above 4 years of age, Hb levels above 9.0 g/dl, transfusion-independent, normal growth, minimal bone changes and slight splenomegaly. Intermediate phenotype included patients diagnosed between 2 ± 4 years, Hb more than 8.0 g/dl, transfusion-independent, with mildly impaired growth, some bone abnormalities and moderate splenomegaly. Severe phenotype included patients with severe anemia, Hb less than, 8.0 g/l, needing frequent or occasional transfusions. Hb H Patients with severe phenotype had impaired growth, moderate to severe bone changes and splenomegaly^7^.

There are two types of Hb H disease, deletional and non-deletional. The first type is the most common form of Hb H disease, is caused by compound heterozygosity with a double α-globin gene deletion on one allele and a single α-globin gene deletion on the other allele (16p13.3). Second type is the non-deletional form of Hb H disease which at least one of the genetic abnormalities was non-deletional^8^. Non-deletional Hb H disease is relatively rare, usually caused by homozygosity for non-deletional alleles, and sometimes by compound heterozygosity for a double α-globin gene deletion on one chromosome and a point mutation of either the α1 or α2 globin gene on the other chromosome^8^. In general, the clinical phenotypes of Hb H disease are variable, ranging from asymptomatic to harsh forms as in some patients on regular/irregular blood transfusion^4^.

Non-deletional Hb H disease has more severe clinical symptoms and they are more anemic, prone to hepatosplenomegaly and transfusion dependency^7,9^. Because of high number of α-thalassemia carriers and consanguineous marriages in Iranian families, the prevalence of individuals with Hb H or even hydrops fetalis is increased^10^.

We evaluated Hb H disease in Iranian patients in Khuzestan Province in order to arrange a sensible prevention and management approach for the disease. Consanguineous and ethnic marriages in this region makes the controlling of disease more complicated and bring the necessity of clinical follow-up and routine screening for anemia at birth, during infancy and childhood.

## Methods

### Patients

We followed medical records of 120 patients with Hb H disease. These patients referred to the Narges Prenatal Diagnostics and Medical Genetics Laboratory as part of a national program for the prevention of thalassemia. Informed consent was obtained from the parents or the patients participating in this study. The red blood cell indices were automatically measured on a Coulter Counter ABX Micros 60 (Helena Laboratories, Beaumont, TX, USA). Hemoglobin H value was measured by high performance liquid chromatography (HPLC) using the VARIANT ™ HPLC system (Bio-Rad Laboratories, Hercules, CA, USA). The HbA2 band was measured by column chromatography (Beta-Thal HbA2 Quik Column Kit, Helena Laboratories) although HbF was performed by hemoglobin electrophoresis on cellulose acetate. Patients came from different cities of Khuzestan province with different ethnics. Phenotypic analysis was performed based on some routine analysis of polar, splenomegaly, Hepatomegaly, transfusion histories, and whether the patient had undergone splenectomy. This study was also reviewed and approved by the Ethics Committee of Pasteur Institute of Iran. All methods were carried out in accordance with relevant guidelines and regulations.

### Genotypic analysis

Molecular studies were conducted on genomic DNA isolated from peripheral blood cells by salting-out procedure^11^. For identifying α-thalassemia genotype, investigation of common mediterranean -globin gene deletions (-^3.7^, -^4.2^ -^20.5^ and --^MED^) was performed by multiplex gap polymerase chain reaction as described previously;^12^ the entire α and β-globin genes was amplified and DNA sequenced, ABI -3130 (Applied Biosystems, Foster City, CA, USA). In order to detect non common alpha deletions, multiplex ligation-dependent probe amplification (MLPA assay) was performed using the SALSA MLPA kit (MRC-Holland, Amsterdam, Netherlands). Then amplified fragments were separated by capillary electrophoresis, on an ABI PRISM 3130 Genetic Analyzer (Applied Biosystems, Foster city, CA, USA) and analysis was performed by gene marker software v.1.6 (Soft Genetics, State College, PA, USA).

## Results

### The cohort

Of the 120 patients with Hb H disease, 50 were male and 70 were female. The average age was 23.0 ± 7 years.

### Hb H genotypes

Different genotype of alpha globin genes was detected among 120 patients in Khuzestan province of Iran. Of these patients, 35 (29.16%) had deletional form of Hb H disease, and 85 (70.83%) had different form of deletional/nondeletional and nondeletional Hb H diseases. The most frequently observed Hb H genotypes were --^Med^/–α^3.7^ in 33 patients (27.5%), α^CD19(-G)^ α^/^α^CD19(-G)^ α in 25 cases (20.83%), α^polyA2^α/α^polyA2^α in 15 (12.5%), and α^polyA1^α/α^polyA1^α in 13 (10.83%) respectively. The probability of receiving at least one transfusion blood in deletional form was observed in 3 of 35(8.57%) patients which just seen in 3 of 33(9%) patients with --^Med^/–α^3.7^ genotype. In deletional/nondeletional and nondeletional forms of Hb H disease blood transfusions was observed in 8 of 85 (9.4%) which included following genotypes, 2 of 2 (100%) --^Med^/α^polyA1^α, 1 of 1 (100%) --^Med^/α^Initiation CD (T>G)^α, 2 of 3 (66.7%) --^Med^/α^CD19(-G)^α , 1 of 2 (50%) α^Constant spring^α/α^Constant spring^α, and 2 of 13 (15.38% ) α^polyA1^α/α^polyA1^α (Table 1).

**Table 1:** Genotype diversity of deletional and nondeletional HbH disease, hematological indices and clinical manifestations.

α-Globin genotype	No. of patients	Transfusion-dependent		Pallor	Splenectomy	Splenomegaly	Hepatomegaly	Ethnic	MCV (mean ± SD)	MCH (mean ± SD)	RBC (mean ± SD)	HbA2 (mean ± SD)	HGB (mean ± SD)	HbH (%)
		No. of patients	Frequency of transfusion											
**Deletional**														
-^Med^/–α^3.7^	33 17 F 16 M	3	1-Monthly, female 2-Two times during pregnancy 3-Once during pregnancy	33	2	10	8	Arab:28 Lor:3 Fars:2	57. 4 ± 6.6	16.62 ± 1.58	5.3 ± 0.75	1.5 ± 0.7	8.3 ± 0.7	7.8 ± 3.7
--^Med^/–α^4.2^	1 F	0	–	1	0	1	1	Arab	55.4	16.2	4.66	1.2	7.5	NR
^-(20.5)^/–α^3.7^	1 F	0	–	1	0	1	1	Fars	64.6	18.6	5.05	0.5	9.4	0.5
**Non-deletional**														
--^Med^/α^poly A6^α	2 F	2	Once	2	2	2	2	Arab:2	56.25 ± 6.2	17.35 ± 1.6	5.38 ± 0.55	1.3 ± 0.14	9.25 ± 0.05	NR
--^Med^/α^poly A4^ α	3 2 F 1 M	0	–	3	0	2	2	Fars:2 Arab:1	56.8 ± 6.3	17 ± 1.75	5.4 ± 0.5	1.1 ± 0.36	9.1 ± 0.15	7.3 ± 0.6
--^Med^/α^CD19(-G)^ α	3 2 F 1 M	2	1-Once after pregnancy 2-Once during pregnancy	3	0	3	3	Arab:3	65.6 ± 4.2	16.6 ± 0.4	4.41 ± 0.2	2 ± 0.2	7.35 ± 0.5	1.5 ± 0.2
--^Med^/α^Initiation CD (T>G)^α	1 F	1	Two times before pregnancy and two times during pregnancy	1	0	1	1	Fars	73	22.8	3.81	1.5	7.5	10
-^(20.5)^/α^5nt^α	1 F	0	-	1	0	1	1	Arab	52.3	15.9	4.65	0.8	7.4	14.3
α^poly A6^α/α^poly A6^α	13 7 F 6 M	2	1-Once during pregnancy 2-Once at age of 40 days	13	0	10	5	Arab:12 Fars:1	62.5 ± 8.7	18.3 ± 3.0	5.09 ± 1.01	2.1 ± 0.7	9.07 ± 2.6	14.8 ± 3.5
α^poly A4^α/α^poly A4^α	15 8 F 7 M	0	–	6	0	2	2	Arab:11 Lor:2 Fars:2	68 ± 2.4	21.07 ± 1.54	5.67 ± 0.6	2.5 ± 0.7	12.0 ± 1.7	NR
α^Constant spring^α/α ^Constant spring^α	2 1 F, 1 M	1	once	2	0	2	2	Lor:1 Shoshtari:1	78.85 ± 3.4	23.65 ± 0.15	4.04 ± 0.09	3.2 ± 2.0	9.55 ± 0.15	0.9 ± 0.1
α^Constant spring^α/α^Hb Icaria^α	1 M	0	–	1	0	1	1	Lor	76.4	22.5	4.58	2.7	10.3	NR
α^poly A6^α/α^Constant spring^α	1 F	0	–	1	0	1	1	Arab	69.28	21.33	5.11	1.5	10.9	NR
α^5nt^ α/α^poly A6^ α	2 F	0	–	0	0	1	1	Arab:2	59.05 ± 1.2	17.9 ± 0.9	6.2 ± 0.3	1.6 ± 1.0	11.15 ± 1.1	NR
α^Initiation CD (T>G)^α/α^Initiation CD (T>G)^α	4 3 M, 1F	0	–	1	0	1	1	Arab:3	64.9 ± 3.2	20.3 ± 1.7	5.66 ± 1.1	2.2 ± 0.8	11.5 ± 1.1	NR
α^5nt^ α/α^5nt^ α	12 7 M, 5 F	0	–	0	0	–	–	Arab:11 Lor:1	64.4 ± 3.7	20.1 ± 1.2	5.6 ± 0.8	2.5 ± 0.56	11.7 ± 1.9	NR
α^CD19(-G)^ α/α^CD19(-G)^ α	25 10 F, 15 M	0	–	0	0	–	–	Arab:12 Lor:13	65.3 ± 3.4	19.9 ± 1.0	5.5 ± 0.7	0.4 ± 0.2	11.3 ± 1.46	NR

*NR* Not reported.

Due to multiethnic nature of khuzestan province like Arabs, lor, Shoshtari, Dezfuli, and Fars, the ethnic background was assessed. The parental ethnic background was Arab in 88 patients (73.33%), Lor in 22 (18.33%), Fars in 9 (7.5%), and Shoshtari in 1 (0.83%).

Arabs in deletional and non-deletional Hb H disease mutations were the dominant ethnic group. The most frequent types of Hb H disease mutations among Arabs was ^Med^/–α^3.7^ in 28 patients (23.3%), α^CD19(-G)^ α^/^α^CD19(-G)^ α in 12 cases (10%), α^polyA1^α/α^polyA1^α in 12 (10%), and α^polyA2^α/α^polyA2^α in 11 (9.16%) respectively (Table 1).

In deletional form of Hb H disease, three patients were received blood who inherited --^Med^/–α^3.7^ genotype. The first patient was a 60 year old woman who was receiving blood every month and who had a hemoglobin level (Hb) of 7.1 g per deciliter (g/dl). She underwent splenectomy because of the need for frequent blood transfusion. Second patient was a 28 year old woman who had Hb of 7.8 g/dl receiving blood for two times during pregnancy and also underwent splenectomy. Third patient was a 24 year old woman who had Hb of 7.9 g/dl receiving blood just once during pregnancy.

In non- deletional form of Hb H disease, eight patients were received blood which five of them had Med deletion in compound with alpha globin point mutations, especially during pregnancy. Interestingly, two patients with the --^Med^/α ^polyA1^α genotype received once transfusion had undergone splenectomy (Table 1).

Hematological parameters were evaluated following genotypes -^(20.5)^/α^5nt^α, --^Med^/–α^4.2^, --^Med^/–α^3.7^, --^Med^/α ^poly A4^ α and --^Med^/α ^poly A6^α were associated with more severe anemia having low hemoglobin level (Hb), mean corpuscular volume (MCV), mean corpuscular hemoglobin (MCH) respectively. The average hematological indices for each genotype are shown in Table 1.

## Discussion

Hb H disease displayed by a varied clinical and hematologic phenotypic heterogeneity which is mostly seen in some regions of Southeast Asia, the Middle East and the Mediterranean countries ^13^. The phenotypic variability of Hb H disease is ranging from asymptomatic, to need for periodic transfusions, to severe anemia with hemolysis^4^.

Of the 120 patients we studied, 29.16% had the deletional genotype and 70.83% had the non-deletional genotype. As in many other genetic diseases, the incidence of Hb H disease varies in different ethnic groups. By contrast with our patients from Khuzestan province, which more ethnic background was Arab (73.33%), the most common α-globin genotype in our Hb H patients was --^Med^/–α^3.7^ (27.5%) that was similar to other reports of Iran and other populations with different ethnic backgrounds^14–19^. This genotype frequency followed by α^CD19(-G)^ α^/^α^CD19(-G)^ α (20.83%), α^polyA2^α/α^polyA2^α (12.5%), and α^polyA1^α/α^polyA1^α (10.83%) genotypes which is almost similar to previous reported study from Khuzestan province^16^. Although, this frequencies of Hb H genotypes are in contrast to Arabian Peninsula countries where the majority of Hb H disease cases are actually due to homozygosity for polyA1 mutation^20^ but of the 13 α^polyA1^α/α^polyA1^α mutations detected 12 were from Arab ethnic group.

In this study we reviewed 12 Hb H genotypes of patients for Hb (g/dl), Hb H (%), transfusion-dependent that presented in our study, Iranian population and other countries. We just selected articles that reported transfusion dependent or independent of Hb H patients. Here, we classified genotypes causing Hb H disease into three Hb H categories: deletional, non-deletional and non deletional/deletional. Of 189 deletional Hb H diseases including -^Med^/–α^3.7^, --^Med^/–α^4.2^ and –(α)^20.5^/–α^3.7^ genotypes, 9 (4.76%) were transfusion-dependent with Hb 9.6 ± 1.8 g/dl and Hb H 8.6 ± 3.5% . In the compound heterozygosity of the deletion and non-deletion genotypes or non deletional/deletional Hb H , seven genotypes as follows: --^Med^/α^CS^α, –(α)^20.5^*/*α^–5nt^α, –(α)^20.5^*/*α^polyA2^α, --^Med^/α^polyA1^α, --^Med^/α^polyA2^α, --^Med^/α^–5nt^α, α^CD19(-G)^ α/--^Med^ were reviewed. Of 93 non deletional/deletional Hb H, 24 (25.8%) were transfusion-dependent, had Hb 8.9 ± 1.1 g/dl and Hb H 14.4 ± 6.2%. In non-deletional Hb H category with α^polyA1^α*/*α^polyA1^α, α^polyA2^α*/*α^polyA2^α and α^CS^α*/*α^CS^α genotypes, 283 Hb H diseases reported, among them 54 (19.8%) had been transfused, had Hb 9.0 ± 1.7 g/dl and Hb H 11.2 ± 4.9%. The most common Hb H genotypes that associated with regular transfusions were --^Med^/α^CS^α and α^CD19(-G)^α/--^Med^ genotypes respectively which is related to non deletional/deletional category (Table 2). Because of large sample size of Med/ –α^3.7^ and α^polyA1^α*/*α^polyA1^α genotypes, we can accurately judge the association of these genotypes with blood receiving. As we showed in Table 2, of 147 ^Med^/–α^3.7^ genotype, 8(5.4%), and of 257 α^polyA1^α*/*α^polyA1^α genotype, 51(19.8%) had been transfused.

**Table 2:** Previous studies reported similar genotypes of our studies from patients with Hb H disease.

Population	α-Genotype	No. of patients	Hb (g/dl) mean ± SD	HB H (%)	Transfusion-dependent (%)			References
					No. of patients (%)	Regular	Irregular	
Iran	-^Med^/–α^3.7^	10	8.5 ± 1.1		3	1	2	^14^
Iran	-^Med^/–α^3.7^	21	7.8 ± 1.1	12.6 ± 3.0	0	0	0	^15^
Iran	-^Med^/–α^3.7^	17	NR	NR	0	0	0	^21^
Cyprus	-^Med^/–α^3.7^	44	9.6 ± 1.15	5.5 ± 3.4	0	0	0	^17^
UAE	-^Med^/–α^3.7^	1	8.2	NR	0	0	0	^22^
Kuwait	-^Med^/–α^3.7^	2	NR	NR	0	0	0	^23^
Oman	-^Med^/–α^3.7^	15	9.6 ± 1.8	NR	2	0	2	^20^
Tunisia	-^Med^/–α^3.7^	4	8.3 ± 0.84	2.7 ± 0.4	0	0	0	^24^
Our study	-^Med^/–α^3.7^	33	8.3 ± 0.7	7.8 ± 3.7	3	1	2	
Total	-^Med^/–α^3.7^	147	8.5 ± 0.8	9.5 ± 3.6	8 (5.4)	2	6	
Iran	--^Med^/–α^4.2^	1	13.6	11.5	0	0	0	^14^
Iran	--^Med^/–α^4.2^	1	13.6	5.3	0	0	0	^15^
Our study	--^Med^/–α^4.2^	1	7.5	NR	0	0	0	
Total	--^Med^/–α^4.2^	3	10.0 ± 4.2	8.4 ± 4.3	0	0	0	
Iran	--^Med^/α^CS^α	3	8.2 ± 0.6	16.4 ± 2.7	2	2	0	^14^
Iran	--^Med^/α^CS^α	4	8.2 ± 0.6	15.3 ± 2.4	2	0	2	^15^
Iran	--^Med^/α^CS^α	3	NR	NR	2	2	0	^21^
Greece	--^Med^/α^CS^α	1	NR	NR	0	0	0	^7^
Total	--^Med^/α^CS^α	11	8.2 ± 0.3	15.8 ± 0.8	6 (54.4)	4	2	
Iran	–(α)^20.5^/–α^3.7^	4	9.9 ± 0.7	10.7 ± 4.0	1	0	1	^14^
Iran	–(α)^20.5^/–α^3.7^	5	10.28 ± 0.7	9.62 ± 0.4	0	0	0	^15^
Iran	–(α)^20.5^/–α^3.7^	11	NR	NR	0	0	0	^21^
Cyprus	–(α)^20.5^/–α^3.7^	12	9.9 ± 1.35	7.2 ± 3.9	0	0	0	^17^
Turkey	–(α)^20.5^/–α^3.7^	6	10.4 ± 1.7	13.5 ± 3.1	0	0	0	^25^
Our study	–(α)^20.5^/–α^3.7^	1	9.4	NR	0	0	0	
Total	–(α)^20.5^/–α^3.7^	39	10.0 ± 0.4	10.2 ± 2.6	1 (2.7)	0	1	
Iran	–(α)^20.5^*/*α^–5nt^α	6	8.6 ± 0.8	15.8 ± 5.3	2	0	2	^14^
Iran	–(α)^20.5^*/*α^–5nt^α	6	8.68 ± 0.8	16.2 ± 5.3	0	0	0	^15^
Turkey	–(α)^20.5^*/*α^–5nt^α	3	8.4 ± 0.5	23.2 ± 9.6	0	0	0	^25^
Our study	–(α)^20.5^*/*α^–5nt^α	1	7.4	14.3	0	0	0	
Total	–(α)^20.5^*/*α^–5nt^α	16	8.3 ± 0.5	17.4 ± 4.0	2 (12.5)	0	2	
Iran	–(α)^20.5^*/*α^polyA2^α	2	9.6 ± 0.5	18.3 ± 4.9	0	0	0	^14^
Iran	–(α)^20.5^*/*α^polyA2^α	3	9.63 ± 0.5	16.2 ± 4.9	0	0	0	^15^
Iran	–(α)^20.5^*/*α^polyA2^α	3	NR	NR	0	0	0	^21^
Cyprus	–(α)^20.5^*/*α^polyA2^α	1	9.5	14.5	0	0	0	^17^
Turkey	–(α)^20.5^*/*α^polyA2^α	1	8.1	12	0	0	0	^25^
Total	–(α)^20.5^*/*α^polyA2^α	10	9.2 ± 0.7	15.2 ± 2.7	0 (0.0)	0	0	
Iran	--^Med^/α^polyA1^α	3	8.7 ± 0.4	8.35 ± 0.7	1	0	1	^15^
Iran	--^Med^/α^polyA1^α	5	NR	NR	3	0	3	^21^
Cyprus	--^Med^/α^polyA1^α	2	9.6 ± 0.28	7.6 ± 0.28	0	0	0	^17^
Kuwait	--^Med^/α^polyA1^α	1	NR	NR	0	0	0	^23^
Our study	--^Med^/α^polyA1^α	2	9.25 ± 0.05	NR	2	0	2	
Total	--^Med^/α^polyA1^α	13	9.2 ± 0.4	8.0 ± 0.5	6 (46.1)	0	6	
Iran	--^Med^/α^polyA2^α	2	10.8 ± 2.0	3.3 ± 0.9	1	0	1	^15^
Iran	--^Med^/α^polyA2^α	2	10.6 ± 0.4	11.5 ± 0.7	2	0	2	^14^
Our study	--^Med^/α^polyA2^α	3	9.1 ± 0.1	7.3 ± 0.6	0	0	0	
Total	--^Med^/α^polyA2^α	7	10.2 ± 0.9	7.4 ± 4.1	3 (42.8)	0	3	
Iran	--^Med^/α^–5nt^α	1	9.9	20.0	0	0	0	^14^
Iran	--^Med^/α^–5nt^α	1	9.9	20.0	1	0	1	^15^
Oman	--^Med^/α^–5nt^α	1	8.5	NR	0	0	0	^20^
Iran	--^Med^/α^5nt^α	1	NR	NR	1	0	1	^21^
Greece	--^Med^/α^5nt^α	8	NR	NR	2	NR	2	^7^
Greece	--^Med^/α^5nt^α	1	7.6	23.7	0	0	0	^26^
Cyprus	--^Med^/α^5nt^α	12	9.3 ± 1.2	21.9 ± 2.3	0	0	0	^17^
Kuwait	--^Med^/α^5nt^α	1	NR	NR	0	0	0	^23^
Total	--^Med^/α^5nt^α	26	9.0 ± 1.1	21.2 ± 2.1	3 (11.5)	0	1	
Iran	α^poly A6^α*/*α^poly A6^α	6	10.38 ± 0.8	12.1 ± 4.2	1	0	1	^15^
Iran	α^poly A6^α*/*α^poly A6^α	2	9.8 ± 0.8	13.0 ± 4.2	1	1	0	^14^
Greece	α^polyA1^α*/*α^polyA1^α	9	NR	NR	3	0	3	^7^
Greece	α^polyA1^α*/*α^polyA1^α	1	7.5	8.0	1	0	1	^26^
Greece	α^polyA1^α*/*α^polyA1^α	3	8.0 ± 0.0	17.0 ± 10.2	0	0	0	^26^
Cyprus	α^poly A6^α*/*α^poly A6^α	3	9.5 ± 0.45	17.3 ± 9.3	0	0	0	^17^
Kuwait	α^poly A6^α*/*α^poly A6^α	133	NR	NR	0	0	0	^23^
Kuwait	α^polyA1^α*/*α^polyA1^α	11	8.98	NR	7	0	7	^27^
UAE	α^poly A6^α*/*α^poly A6^α	6	8.3	NR	1	1	0	^22^
Oman	α^polyA1^α*/*α^polyA1^α	25	9.1 ± 1.7	NR	10	2	8	^20^
Tunisia	α^polyA1^α*/*α^polyA1^α	6	8.5 ± 0.65	8.0 ± 0.8	1	0	1	^24^
Bahrain	α^polyA1^α*/*α^polyA1^α	32	8.5 ± 0.7	NR	23	0	23	^28^
Turkey	α^polyA1^α*/*α^polyA1^α	7	8.41 ± 1.2	15	1	0	1	^29^
Our study	α^polyA1^α*/*α^polyA1^α	13	9.07 ± 2.6	14.8 ± 3.5	2	0	2	
Total	α^polyA1^α*/*α^polyA1^α	257	8.8 ± 0.8	13.1 ± 3.6	51 (19.8)	4	47	
Iran	α^polyA2^α*/*α^polyA2^α	3	10.7 ± 2.6	11.0 ± 1.2	0	0	0	^14^
Turkey	α^polyA2^α*/*α^polyA2^α	2	6.8 ± 0.14	14.05 ± 8	1	NR	1	^25^
Our study	α^polyA2^α*/*α^polyA2^α	15	12.0 ± 1.7	NR	0	0	0	
Total	α^polyA2^α*/*α^polyA2^α	20	9.8 ± 2.7	12.5 ± 2.1	1 (5.0)	0	1	
Iran	α^CS^α*/*α^CS^α	1	5.3	10.6	1	0	1	^14^
Iran	α^CS^α*/*α^CS^α	1	12	4.1	0	0	0	^15^
UAE	α^CS^α*/*α^CS^α	2	10	NR	0	0	0	^22^
Thailand	α^CS^α*/*α^CS^α	16	10.3 ± 1.0	NR	6	0	6	^30^
Our study	α^CS^α*/*α^CS^α	2	9.5 ± 0.1	0.9 ± 0.1	1	0	1	
Total	α^CS^α*/*α^CS^α	22	9.4 ± 2.5	5.2 ± 4.9	8 (36.3)	0	8	
Iran	α^CD19(-G)^ α/--^Med^	1	10.7	16.5	0	0	0	^15^
Kuwait	α^CD19(-G)^ α/--^Med^	2	6.0	NR	2	2	0	^23^
Kuwait	α^CD19(-G)^ α/--^Med^	4	NR	NR	0	0	0	^23^
Our study	α^CD19(-G)^ α/--^Med^	3	7.35 ± 0.5	1.5 ± 0.2	2	0	2	
Total	α^CD19(-G)^ α/--^Med^	10	8.0 ± 2.4	9.0 ± 10.6	4 (40)	2	2	

*NR* Not reported.

It seems that the clinical phenotype of ^Med^/–α^3.7^ genotype usually presents with mild or moderate thalassemia. The probable cause of blood transfusion receiving in some patients might be a consequence of circumstantial factors or other modifying factors that play a role in the proteolytic capacities of the erythroid cells^21^.

On the other hand, red cells hemolysis in Hb Constant Spring is maybe because of precipitation and aggregation of mRNAs that affecting the red cell membrane and producing visible basophilic stippling^30^.

According to this study, patients with non deletional/deletional and nondeletional Hb H disease usually are more anemic, and more likely to require transfusions suggested that Hb H disease is not as benign a disorder.

The diagnosis of Hb H disease at the molecular level is important for genetic counseling and the identification of families at risk for having pregnancies affected with Hb H disease. Regarding the need for blood transfusion in deletional and non- deletional Hb H disease, most of deletional Hb H cases were managed without blood transfusion. Nondeletional Hb H disease was more severe than deletional Hb H disease, with patients undergoing lower Hb levels and higher HB H percentage, requiring more blood transfusions and should be monitored closely. Therefore, we can recommend that Med deletion in compound with alpha-globin point mutations, polyA1 and constant spring in homozygous form needs to be taken into consideration when offering counseling to high-risk couples.

## Data Availability

All data generated during and/or analyzed during the current study are available upon request by contact the corresponding author.
